# Advanced Large-Stokes-Shift Fluorescent Probe for the Detection of Biothiols: Facilitating Accurate Indirect Measurement of β-Lactamases

**DOI:** 10.3390/ijms26020525

**Published:** 2025-01-09

**Authors:** Likun Liu, Dongling Yan, Yukun Ma, Peng Hou, Pengfei Qi, Xue Zhang, Yitong Liu, Song Chen

**Affiliations:** 1 Research Institute of Medicine & Pharmacy, Qiqihar Medical University, Qiqihar 161006, China; 2 College of Pharmacy, Qiqihar Medical University, Qiqihar 161006, China

**Keywords:** fluorescent probe, biothiols, β-lactamase, inhibitor, antibiotic-resistant bacteria

## Abstract

A novel fluorescent probe, Bibc-DNBS, based on the combination of the PET (photoinduced electron transfer) and ESIPT (excited-state intramolecular proton transfer) mechanisms, was designed and synthesized. Bibc-DNBS exhibited a Stokes shift of 172 nm in the fluorescence detection field. In addition, the probe exhibited good performance in key parameters in bioassays such as sensitivity, specificity, and response time. Based on these properties, Bibc-DNBS successfully monitored the biothiol levels in live cells and zebrafish models, providing an effective analytical tool for real-time monitoring of biothiols. More importantly, Bibc-DNBS could be useful for indirectly detecting β-lactamases. Bibc-DNBS(3-(1H-benzo[d]imidazol-2-yl)-4′-cyano-[1,1′-biphenyl]-4-yl2,4-dinitrobenzenesulfonate) facilitated the screening of β-lactamase inhibitors, using tazobactam and clavulanic acid as model compounds, with respective semi-inhibitory concentration values of 31.32 μM and 2.26 μM, respectively. It might also be applied to distinguish sensitive strain *Staphylococcus aureus* ATCC 29213 and drug-resistant strain *Enterobacter cloacae* ATCC 13047, which could provide strong support for the clinical application of antibiotics and the development of new drugs.

## 1. Introduction

β-lactam antibiotics, including penicillins, cephalosporins, and carbapenems, play an irreplaceable role in the treatment of bacterial infectious diseases by effectively killing or inhibiting bacterial growth, and by providing vital support to the human immune system in fighting infections and promoting healing processes [1]. However, as the evolution of bacteria and the overuse of antibiotics have led to the development of bacterial resistance, the threat of superbugs could cause up to 10 million deaths per year globally by the upcoming year of 2050, even exceeding the number of deaths caused by cancer. This grim prediction highlights the serious challenges currently faced by global public health [2]. An important mechanism for antibiotic resistance is reported to be the production of β-lactamases by bacteria that hydrolyze β-lactam antibiotics into inactivated forms, leading to a high degree of bacterial resistance to almost all β-lactam antibiotics [3]. Clinical studies have demonstrated that over 80% of drug-resistant bacteria exhibit resistance due to the production of β-lactamase [4]. Therefore, detecting β-lactamase is an important step in determining whether bacteria are resistant to β-lactam antibiotics and can significantly contribute to selecting the most suitable antibiotic for patients.

In the study of bacterial resistance, in addition to the action of β-lactamases, bacteria produce large amounts of reactive oxygen species (ROS) under antibiotic stress, leading to oxidative stress. Biothiols mainly consist of homocysteine (Hcy), glutathione (GSH), and cysteine (Cys), which foster a moderate buffer environment in organisms, maintaining redox balance in physiological and pathological processes [5,6,7]. Not only do biothiols play a central role in the antioxidant defense of cells but they may also influence the resistance of bacteria to antibiotics by regulating their oxidative stress response. Therefore, the role of biothiols in antibiotic resistance is an area that deserves in-depth study, and they may provide new ideas for the development of new therapeutic strategies and drugs. Furthermore, abnormal levels of Cys can be associated with conditions like homocystinuria and kidney dysfunction [8,9,10]. Elevated levels of Hcy indicate an increased risk of cardiovascular disease, stroke, and other vascular conditions [11,12]. Changes in GSH levels are linked to oxidative stress and various diseases, such as neurodegenerative disorders, cardiovascular diseases, and cancer [13,14]. Therefore, monitoring the fluctuations in these sulfur-containing compounds can provide valuable insights for assessing an individual’s health status and contribute to the early diagnosis of certain diseases [15,16,17,18,19]. In recent years, the interest in biothiol determination has intensified significantly, driven by the crucial roles these molecules play in maintaining redox balance, combating oxidative stress, and regulating numerous biological processes. To this end, researchers have not only refined traditional methods such as electrochemical analysis [20], colorimetric methods [21], and high-performance liquid chromatography [22] but have also ventured into innovative avenues like capillary electrophoresis [23] and advanced optical detection techniques [24]. These newer methods offer sensitive, selective, and rapid quantification and qualification of biothiols, enabling deeper insights into their dynamic behavior under various physiological and pathological conditions (Table S1). These advancements have not only broadened our understanding of biothiol function and the regulatory mechanisms in biology but also paved the way for their potential application in disease diagnosis, prognosis assessment, and therapeutic interventions.

**Scheme 1:**
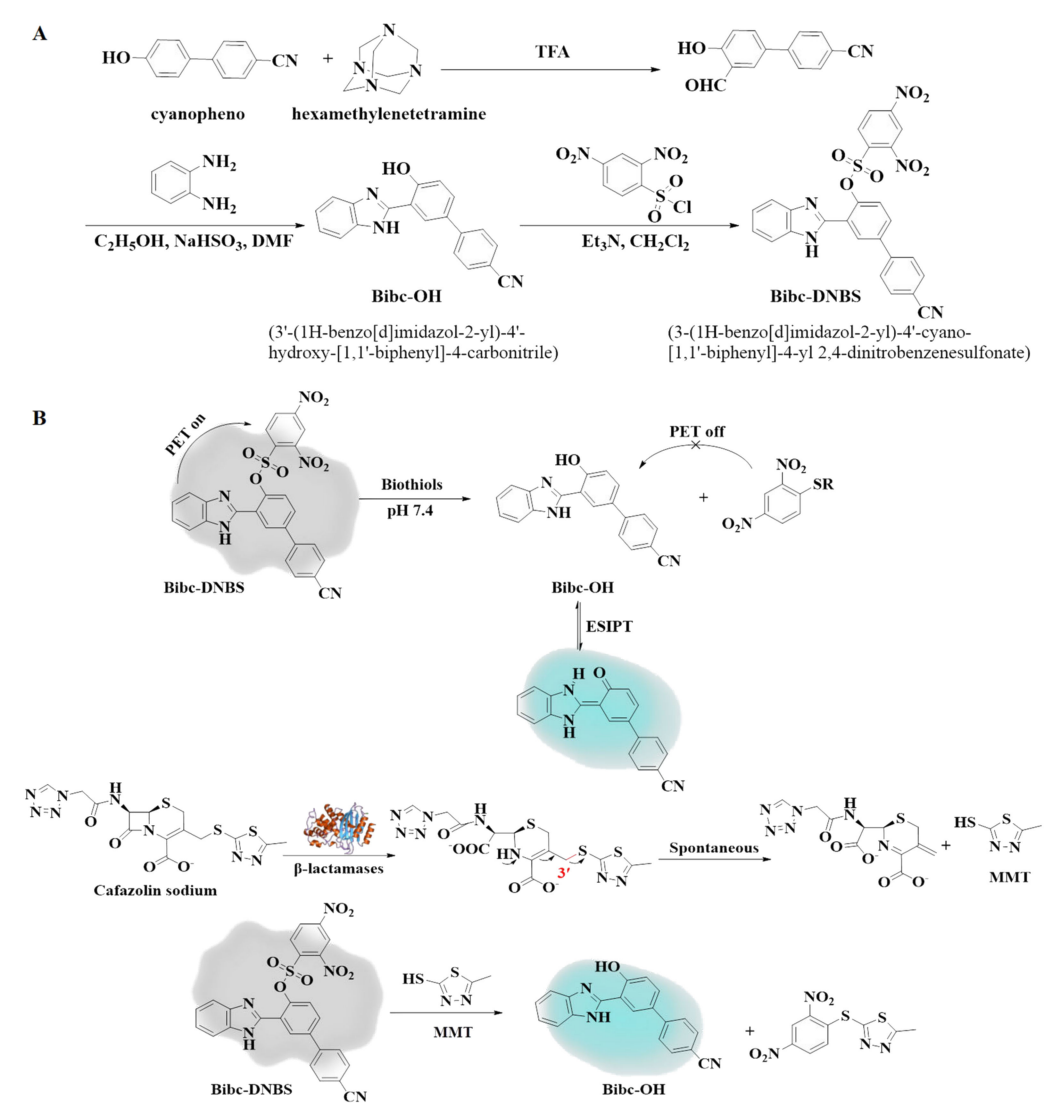
(**A**) Synthetic route to Bibc-DNBS. (**B**) Mechanism of thiol recognition and the indirect detection of β-lactamase by Bibc-DNBS.

Fluorescent probes, renowned for their simplicity, cost-efficiency, remarkable sensitivity, and real-time detection prowess, have garnered significant attention for their pivotal role in facilitating effective in vitro and in vivo studies across a wide range of analytes [25,26,27,28,29]. Currently, the fluorescent probes for β-lactamases were modified based on cephalosporins, which take advantage of the hydrolyzing properties of β-lactamases towards cephalosporins to achieve detection. It is important to note that, while these probes can be effective for detecting β-lactamase activity, they often require complex synthesis processes and intricate modifications of the substrate (β-lactam ring) [30,31]. The specific structural requirements make the design and synthesis of β-lactamase probes more challenging. Recently, it has been found that β-lactamases can disrupt the β-lactam ring of cephalosporin antibiotics and hydrolyze to produce compounds with thiol groups [32]. In this study, we have explored and synthesized a novel fluorescent probe, Bibc-DNBS. Firstly, based on cyanophenol [33], a widely used fluorophore, a fluorescent core structure based on excited-state intramolecular proton transfer (ESIPT) was elaborately designed and synthesized by strategically introducing hexamethylenetetramine, achieving a significant redshift of the emission wavelength and a large Stokes shift of 172 nm (Table S2), which significantly reduced the background interference and improved the signal contrast, thus enhancing the accuracy and reliability of detection to a certain extent, which is of great significance in the field of bioimaging and diagnostics [34]. To enhance the selectivity and sensitivity, we incorporated 2,4-dinitrobenzenesulfonyl chloride as the responsive site for thiols [35], obtaining the Bibc-DNBS probe (Scheme 1A). Bibc-DNBS is based on dual regulation of ESIPT and PET (photoinduced electron transfer). As the core of Bibc-DNBS, the fluorescent signal reporter group Bibc-OH generates detectable fluorescent signals and the responsive site 2,4-dinitrobenzenesulfonate (DNBS), which can effectively quench the fluorescence of Bibc-OH through the PET process. When Bibc-DNBS encounters thiols, the strong nucleophilicity of thiols triggers a specific thiolysis reaction (the reaction of biothiols with thioester bonds of fluorescent probe Bibc-DNBS to restore or alter fluorescence signals) with DNBS, leading to the detachment of the DNBS group from Bibc-OH. This reaction not only restores the ESIPT process of Bibc-OH but also eliminates the quenching effect of the PET process on fluorescence, resulting in a significant enhancement in the fluorescent signal (Scheme 1B). Therefore, by monitoring the changes in fluorescence intensity, detection of biothiols and β-lactamases can be achieved, which is essential for understanding the role of biothiols in redox homeostasis and bacterial drug resistance.

In view of the close connection between biothiol concentration and various pathological states, and the centrality of β-lactamase detection in the evaluation of bacterial drug resistance mechanisms, this study strategically developed Bibc-DNBS, a fluorescent probe with large Stokes shift, whose unique structure and properties not only demonstrate our innovative ability in the field of fluorescent probes but also show great application in the detection of biothiols. The Bibc-DNBS probe is designed to facilitate early warning of diseases by accurately monitoring biothiols and β-lactamases, and to provide a scientific basis for rational selection of antibiotics in clinical settings.

## 2. Results and Discussion

### 2.1. Spectral Responses to Biothiols

**Figure 1:**
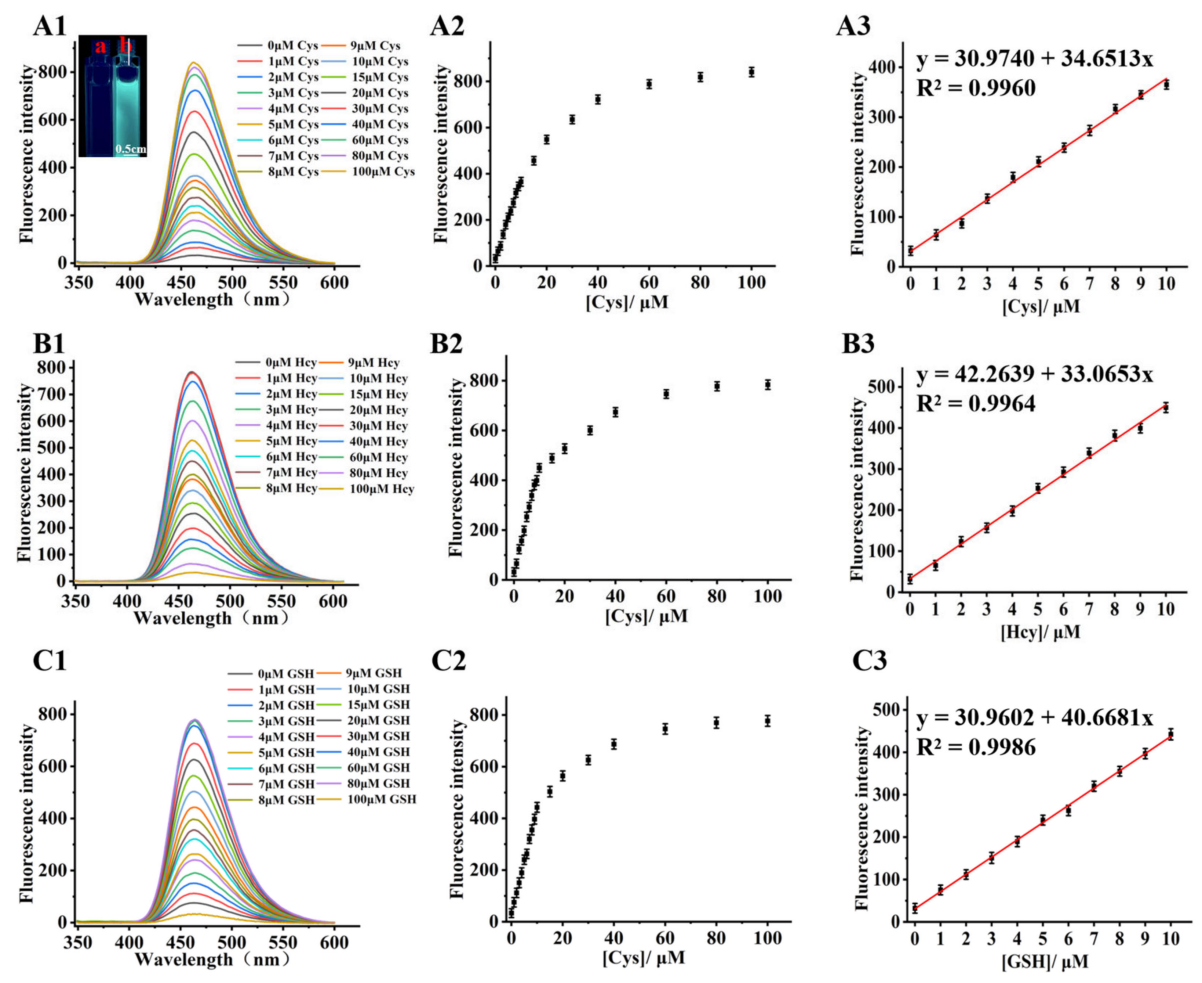
Fluorescence changes in the fluorescent probe Bibc-DNBS (10.0 μM) interacting with 0.0–100.0 μM Cys (cysteine, **A**)/Hcy (homocysteine, **B**)/GSH (glutathione, **C**). (**1**) Fluorescence spectrogram; (**2**) fluorescence intensity versus Cys/Hcy/GSH concentration; (**3**) linear fit of fluorescence intensity to (0.0–10.0 μM) Cys/Hcy/GSH. Inset: cuvette image of probe Bibc-DNBS before (a) and after reaction (b) with Cys taken under a handheld UV lamp (λ_ex_ = 365 nm), scale bar 0.5 cm.

All the spectra detection processes were carried out in acetonitrile/PBS (*v*/*v* = 2/8, pH = 7.4). The fluorescence spectra of Bibc-DNBS treated with a series of biothiols (0.0–100.0 μM) were measured in Figure 1. The probe Bibc-DNBS (10.0 μM) had weak fluorescence emission. With the addition of biothiols, the fluorescence intensity at 462 nm increased about 26/24/23-fold for Cys/Hcy/GSH (Figure 1A1,A2,B1,B2,C1,C2), correspondingly. There was a well-defined linear relationship between the intensity enhancement and the concentration of biothiols (LOD of 0.042/0.092/0.121 μM for Cys/Hcy/GSH) (Figure 1A3,B3,C3). In contrast, there were no obvious changes in the emission spectra of Bibc-DNBS upon introduction of possible competing essential amino acids (Phenylalanine (Phe), Alanine (Ala), Tryptophan (Trp), Histidin (His), Lysine (Lys), Proline (Pro), Methionine (Met), Arginine (Arg), Aspartic acid (Asp), Glutamic acid (Glu), Threonine (Thr), Valine (Val), Isoleucine (Ile)), several ions (Mn^2+^, Na^+^, Mg^2+^, AcO^−^, NO_2_^−^, SO_3_^2−^, and PO_4_^3−^), and other analytes (glucose and citric acid). Moreover, the sensing ability of Bibc-DNBS to biothiols was assessed in the presence of other interfering substances. As illustrated in Figure S1, there was no remarkable influence on recognizing biothiols in aqueous solutions. The stable behavior of Bibc-DNBS and satisfactory fluorescence response at pH values between 6 and 8 (Figure S2) and at temperatures between 32–40 °C (Figure S3) ensured the feasibility of the physiological state for biothiol detection. Meanwhile, it was found that the fluorescence intensity increased continuously and reached saturation within 300 s upon addition of Cys, Hcy, or GSH, respectively (Figure S4), and maintained a stable fluorescence turn-on for the subsequent 8 h (Figure S5). All these phenomena showed that Bibc-DNBS could be used as an efficacious candidate for real-time sensing of biothiols.

### 2.2. HepG2 Cells and Zebrafish Imaging

**Figure 2:**
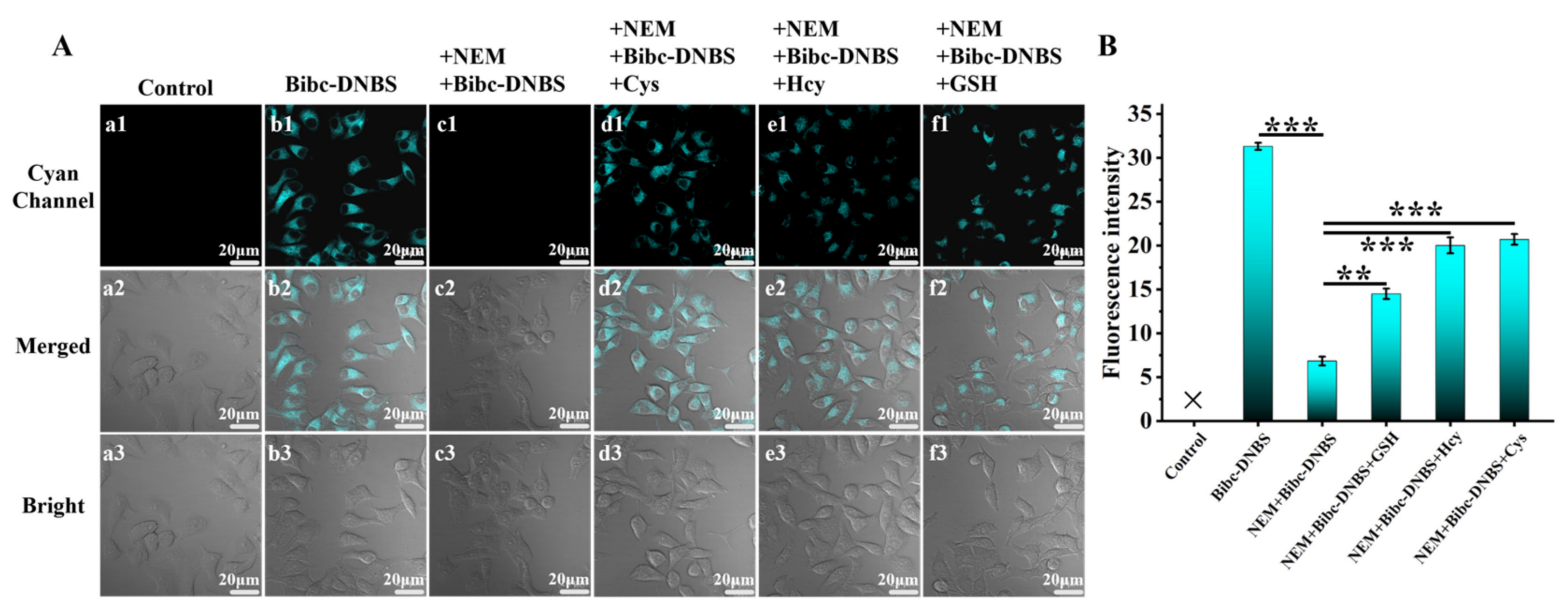
(**A**) Detection of thiols in HepG2 cells by probe Bibc-DNBS confocal imaging. (**a1**–**a3**) Control: HepG2 cells not incubated with the probe Bibc-DNBS. (**b1**–**b3**) HepG2 cells were incubated with the probe Bibc-DNBS (10.0 μM) for 30 min. (**c1**–**c3**) HepG2 cells were first stimulated with NEM (N-ethylmaleimide, 1.0 mM) for 30 min, followed by incubation with Bibc-DNBS (10.0 μM) for an additional 30 min. (**d1**–**d3**) NEM-HepG2 cells were treated with 100.0 μM Cys, (**e1**–**e3**) 100.0 μM Hcy, (**f1**–**f3**) 100.0 μM GSH for 30 min, and incubated with 10.0 μM Bibc-DNBS for additional 30 min, respectively. (**B**) Fluorescence intensities in panels (**a**–**f**). n = 3; error bars are ±SD. Statistical analysis was performed with a one-way ANOVA. Among them, ** *p* < 0.01, *** *p* < 0.001. Scale bar 20 μm.

**Figure 3:**
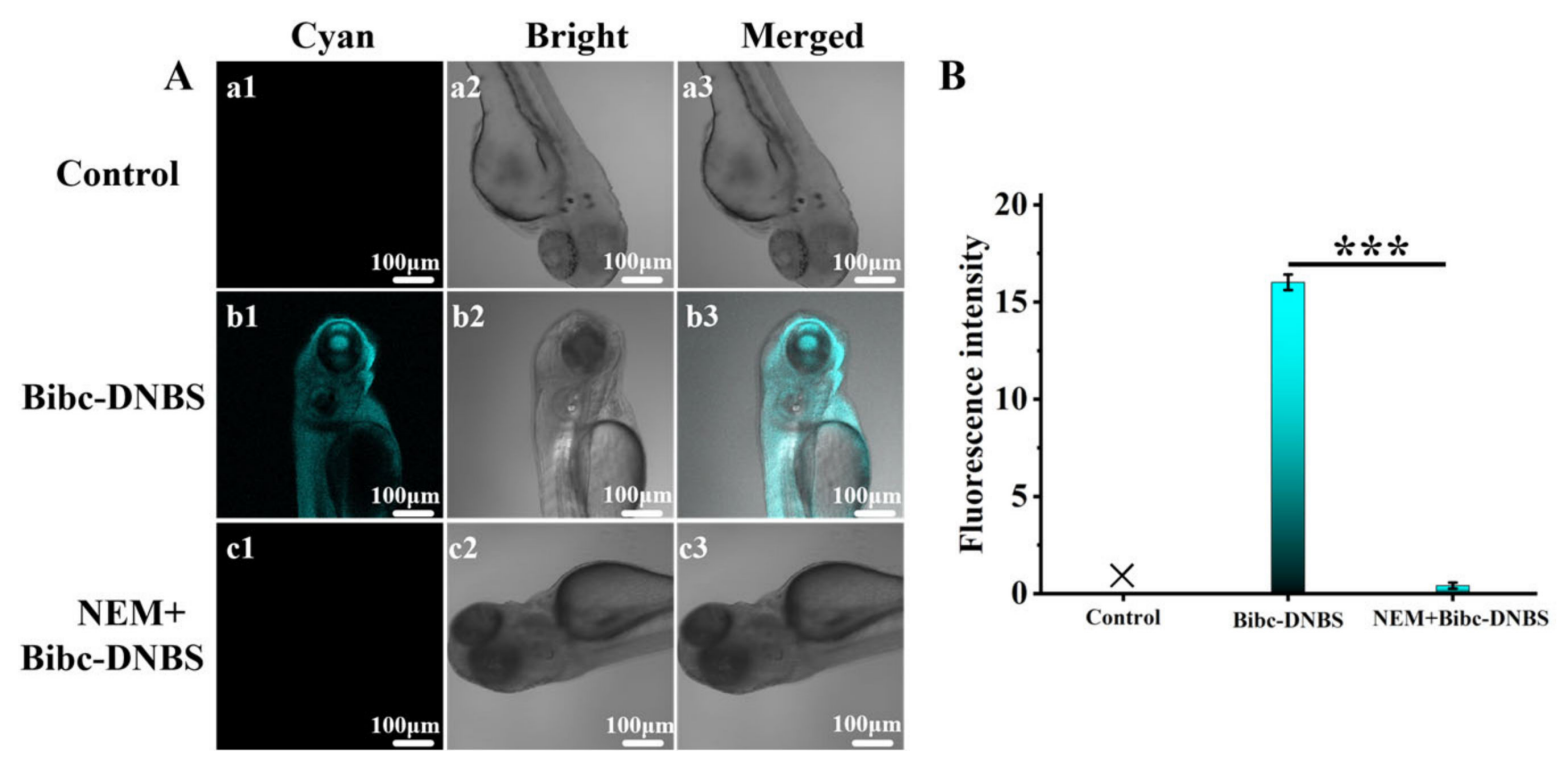
(**A**) Imaging experiments for detection of thiols in zebrafish. (**a1**–**a3**) Control: zebrafish not incubated with the probe Bibc-DNBS. (**b1**–**b3**) Zebrafish was incubated with Bibc-DNBS (10.0 μM) for 30 min. (**c1**–**c3**) Zebrafish was pretreated with NEM (1.0 mM) for 30 min, then incubated with probe Bibc-DNBS (10.0 μM) for another 30 min. (**B**) Fluorescence intensities in panels (**a**–**c**). n = 3; error bars are ±SD. Statistical analysis was performed with a one-way ANOVA. Among them, *** *p* < 0.001. Scale bar 100 μm.

For organism carriers, Bibc-DNBS exhibited relatively low cytotoxicity, with more than a 90% survival rate in HepG2 cells (Figure S6). As shown in Figure 2, bright cyan fluorescence was observed when HepG2 cells were stained via Bibc-DNBS incubation. In stark contrast, the fluorescence signal was greatly weakened with the addition of the thiolblocking reagent (N-ethylmaleimide, NEM). Subsequently, the intense cyan fluorescence of Bibc-DNBS appeared again when NEM-HepG2 cells were supplemented with Cys/Hcy/GSH (100.0 μM). Additionally, similar satisfactory results were obtained from zebrafish imaging (Figure 3). It was observed that there were significantly different fluorescence behaviors in zebrafish with and without NEM administration. This indicates that Bibc-DNBS has good tissue penetration and can potentially serve as a molecular tool for the detection of biothiols in vivo.

Based on the critical role of biothiols in maintaining redox homeostasis in physiological and pathological processes, the HepG2 cell model was employed and the changes in biothiol concentration were systematically analyzed under the LPS-induced oxidative stress environment. The experimental results indicate that the intracellular biothiol concentration demonstrated a gradual decrease with the increase in reactive oxygen species (ROS) concentration, and this phenomenon was reflected in the decrease in fluorescence intensity (Figure S7). This suggests that the probe Bibc-DNBS is able to indirectly monitor the level of intracellular oxidative stress by detecting changes in fluorescence intensity.

### 2.3. Optical Responses of Bibc-DNBS Towards β-Lactamase

**Figure 4:**
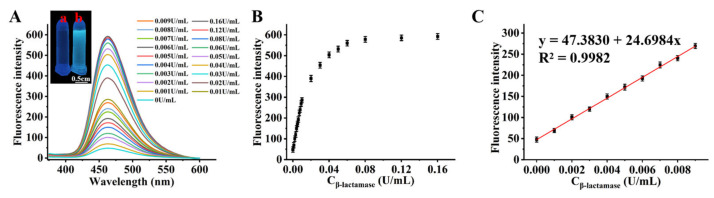
(**A**) Fluorescence spectra of Bibc-DNBS upon addition of different concentrations of β-lactamase (0–0.16 U/mL) containing 250.0 μM cefazolin sodium. Inset: cuvette image of probe Bibc-DNBS before (a) and after reaction (b) with β-lactamase taken under a handheld UV lamp (λ_ex_ = 365 nm), scale bar 0.5 cm. (**B**) With the concentration of β-lactamase being (0.0–0.16 U/mL), the fluorescence signal intensity scattering diagram of Bibc-DNBS (10.0 μM) at 462 nm. (**C**) Linear diagram of fluorescence signal intensity of probe and β-lactamase (0.000–0.010 U/mL).

Using cefazolin sodium as a substrate, the fluorescence spectral characteristics of the Bibc-DNBS fluorescent probe were investigated in the presence and absence of β-lactamase. The testing solution containing Bibc-DNBS and cefazolin sodium was almost non-fluorescent at 462 nm without β-lactamase. However, the fluorescence intensity was progressively enhanced with the increase in β-lactamase concentration (Figure 4A). Compared to the β-lactamase-free solution, the fluorescence intensity at 462 nm was enhanced by 12-fold (Figure 4B). The reaction resulted in cyan fluorescence, which was clearly identified via the naked eye against colorless under UV light irradiation (365 nm) (Figure S8). Such fluorescence enhancement for the assay system after the addition of β-lactamase is owed to the release of the MMT unit in cefazolin sodium and generation of fluorophore Bibc-OH, as discussed above. As observed, the fluorescence intensity possessed a good linear correlation with the β-lactamase concentration (0.0–0.01 U/mL) (y= 47.3830 + 24.6984x, R^2^ = 0.9982) (Figure 4C). The detection limit was determined to be 1.8 × 10^−5^ U/mL based on the LOD = 3δ/k. The excellent alteration in fluorescence spectra attested that Bibc-DNBS could indirectly detect lower concentrations of β-lactamase, which provided the possibility of sensitive screening of enzyme inhibitors.

The fluorescence spectra of Bibc-DNBS for β-lactamase in different pH aqueous solutions were investigated. Without β-lactamase, no significant intensity changes were observed. Bibc-DNBS showed excellent stability under a wide range of pH conditions. For comparison, Bibc-DNBS exhibited a wide range of positive fluorescence responses towards β-lactamase between pH values of 6.0 and 8.0 (Figure S9). The outstanding enhancement indicated that Bibc-DNBS has the potential to detect β-lactamases under physiological conditions. Within 240 s of dynamic measurement, the fluorescence intensity exhibited a normal upward trend in correlation with the β-lactamase content, ranging from 0 to 0.16 U/mL. (Figure S10). When the β-lactamase concentration reached 0.16, a reaction saturation time of 120 s was achieved, suggesting that the fluorescent Bibc-DNBS at a concentration of 10.0 μM has the potential to enable rapid, real-time, and indirect determination of β-lactamase activity.

### 2.4. Mechanism Studies

**Figure 5:**
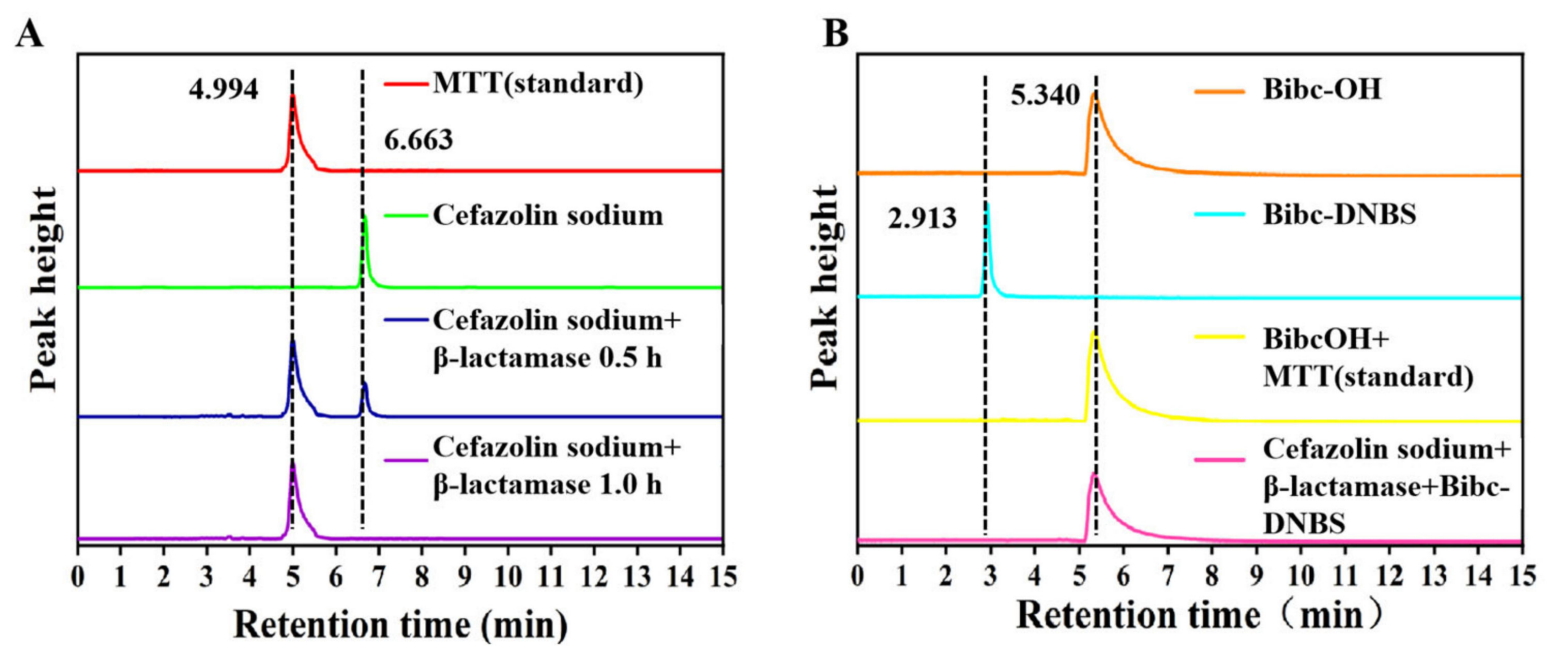
(**A**) HPLC chromatogram of the reaction of cefazolin sodium with β-lactamase: (red) MMT standard, (green) cefazolin sodium, (blue) cefazolin sodium reacted with beta-lactamase for 0.5 h, and (purple) cefazolin sodium reacted with beta-lactamase for 1 h. (**B**) HPLC chromatogram of Bibc-DNBS reacting with β-lactamase: (orange) Bibc-OH, (cyan) Bibc-DNBS, (yellow) Bibc-DNBS reacted with MMT standard, and (pink) cefazolin sodium reacted with β-lactamase and Bibc-DNBS. Conditions: eluent H_2_O/CH_3_CN (*v*/*v*, 90/10); flow rate 1.0 mL/min; temperature 30 °C; sample injection volume 20.0 μL.

To validate the sensing mechanisms, we investigated how the introduction of β-lactamase splits the β-lactam ring in cefazolin sodium to produce the compound MMT containing a sulfydryl group, which further undergoes a nucleophilic substitution reaction with the Bibc-DNBS to release the compound Bibc-OH with strong cyan fluorescence, thus achieving the indirect detection of β-lactamase. The data of high-performance liquid chromatography (HPLC) for the corresponding compounds were determined (Figure 5). The results reveal retention times of 6.663 min and 4.994 min for cefazolin sodium and compound MMT. A new 4.994 min peak appeared when cefazolin sodium and β-lactamase were mixed, which is consistent with the characteristic peak of compound MMT. It was demonstrated that β-lactamase could cleave the β-lactam ring in cefazolin sodium to produce compound MMT (Figure 5A). Meanwhile, the retention time presented by the reaction of Bibc-DNBS with compound MMT coincided with the retention time of compound Bibc-OH (5.340 min), meaning that Bibc-DNBS triggered compound MMT to relax fluorophore Bibc-OH (Figure 5B). In addition, the absorption peak generated by Bibc-DNBS mixed with cefazolin sodium and β-lactamase overlapped with the retention time of Bibc-OH, further confirming that Bibc-DNBS could effectively realize the indirect detection of β-lactamase by capturing the sulfhydryl-containing product MMT. It is worth mentioning that the detection mechanism of Bibc-DNBS for biothiols was rigorously verified with the help of high-performance liquid chromatography (Figure S11) and mass spectrometry (Figure S12), which proved the detection mechanism of Bibc-DNBS for the specific recognition of biothiols.

### 2.5. β-Lactamase Inhibitor Screening

Screening for β-lactamase inhibitors is a vital strategy in improving the efficacy of antibiotics and addressing the challenge of antibiotic resistance. To confirm the capability of Bibc-DNBS in screening β-lactamase inhibitors, we selected clavulanic acid and tazobactam as test subjects and conducted a detailed study on their inhibitory effects. Due to the irreversible binding towards β-lactamase, its content was consumed by inhibitors, resulting in a decrease in the generation of compound MMT. Specifically, 1.0 μM and 16.0 μM of tazobactam inhibited the β-lactamase activity by 28% and 95%, respectively, and 1.0 μM and 16.0 μM of clavulanic acid inhibited 10% and 41% of the β-lactamase activity. The inhibitory efficiency of tazobactam was superior to that of clavulanic acid, which agreed with previous reports [36]. The semi-inhibitory concentrations of clavulanic acid and tazobactam to β-lactamase were evaluated to be 31.32 μM and 2.26 μM, as shown in Figure S13. These results demonstrate the great potential and sensitivity of Bibc-DNBS employed to sift β-lactamase inhibitors.

### 2.6. Drug Resistance

**Figure 6:**
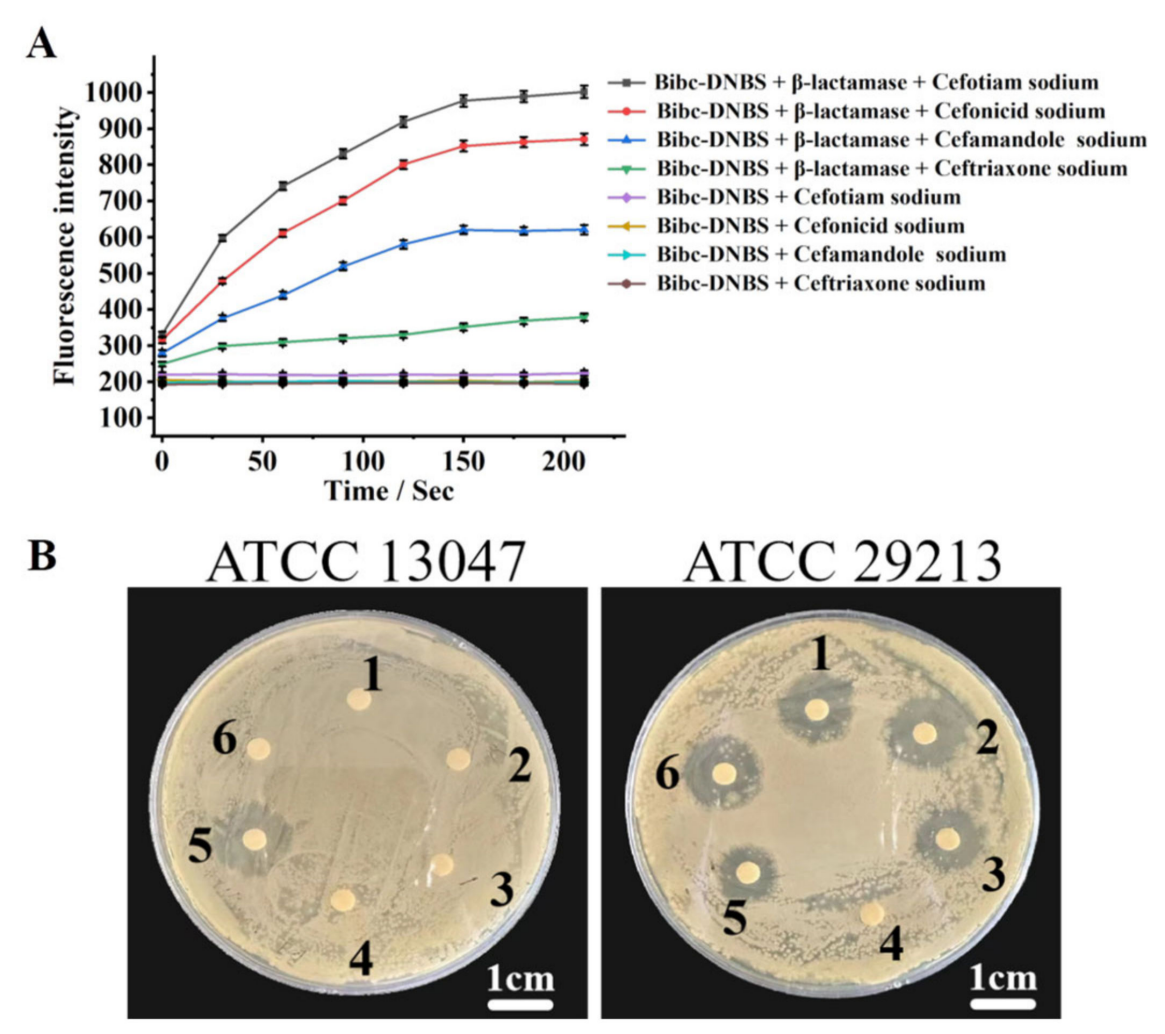
(**A**) The fluorescence intensity of Bibc-DNBS with or without β-lactamase was recorded by enzyme-labeling instrument when different antibiotics were used as substrates. (**B**) Inhibition circle test (1–6 cefazolin sodium, cefotiam sodium, cefonicid sodium, blank reagents, ceftriaxone sodium, and cefamandole sodium); scale bar 1 cm.

Bibc-DNBS can also be utilized to estimate drug resistance. Cefotiam, cefonicid, cefamandole, ceftriaxone, and cefazolin sodium were used as substrates to interact with β-lactamases. As illustrated in Figure 6A, the various antibiotics were unable to hydrolyze the target thiol-containing compounds in the absence of β-lactamase. There was little change in the fluorescence intensity due to the lack of hydrolysis products interacting with Bibc-DNBS. Upon the treatment of β-lactamase, the antibiotic would be hydrolyzed with β-lactamase to produce MMT with sulfhydryl groups. The fluorescence intensity prominently ascended in the assay system of Bibc-DNBS. In addition, research has found that the fluorescence feedback performance of Bibc-DNBS was much stronger than that of ceftriaxone after reacting with β-lactamases in cefotaxime, cefonidib, and cefomandol. This clearly indicates that, compared to ceftriaxone, β-lactamases demonstrate faster hydrolysis efficiency for the three drugs. This phenomenon may be attributed to the stronger anti-enzyme properties of ceftriaxone, which reduces its susceptibility to β-lactamases.

To further demonstrate the effectiveness of this analysis method in evaluating drug resistance, the inhibition zones in the cephalosporin drugs (cefotiam, cefonicid, cefamandole, ceftriaxone, and cefazolin sodium) of *Staphylococcus aureus* ATCC 29213 and *Enterobacter cloacae* ATCC 13047 were obtained (Figure 6B). The images indicate that obvious antibacterial zones emerged in the antibiotic-sensitive strain (*Staphylococcus aureus* ATCC 29213) through inhibition of cefotiam, cefonicid, cefamandole, ceftriaxone, and cefazolin sodium. In stark contrast, there was one antibacterial zone of *Enterobacter cloacae* ATCC 13047 in ceftriaxone, whereas the antibacterial phenomenon disappeared in the areas of cefotiam, cefonicid, cefamandole, and cefazolin sodium. This phenomenon occurred because cefotiam, cefonicid, cefamandole, and cefazolin sodium were rapidly resisted and completely hydrolyzed by the β-lactamase released by *Enterobacter cloacae* ATCC 13047. However, the hydrolysis rate of ceftriaxone with β-lactamase was slower, enabling the observation of an inhibition zone in strains produced with β-lactamase, which was in agreement with the above evaluation of hydrolysis efficiency. These results demonstrate that the assay system of Bibc-DNBS can be exploited to appraise the competence of antibiotics against resistant bacteria.

## 3. Materials and Methods

### 3.1. Materials and Instruments

All reagents were purchased from commercial companies and could be used without further purification. Both NMR data of Bibc-DNBS and Bibc-OH were characterized by a Bruker Avance 600 MHz spectrometer (Bruker Corporation, Billerica, MA, USA). High-resolution mass spectra were obtained with a Thermo Scientific LTQ Orbitrap XL FTMS (Thermo Fisher Scientific, Waltham, MA, USA). Cell imaging was performed on a Zeiss LSM710 Wetzlar (Carl Zeiss AG, Oberkochen, Germany) confocal laser scanning microscope. Fluorescence spectra were measured with a HITACHI F-4600 (Hitachi High-Technologies Corporation, Tokyo, Japan) fluorescence spectrophotometer. The determination of β-lactamase was carried out with Multifunctional Enzyme Labeler (EnSpire-2300 (PerkinElmer Inc., Waltham, MA, USA)).

### 3.2. Synthesis of Bibc-OH

Further, 22.3 mg (0.1 mM) of 3′-formyl-4′-hydroxy-[1,1′-biphenyl]-4-carbonitrile and NaHSO_3_ (12.5 mg, 0.12 mM) were mixed in a round-bottomed flask containing 10 mL of anhydrous ethanol and refluxed at 80 °C for 3 h. Then, 3.0 mL of DMF and 10.8 mg (0.1 mM) of O-phenylenediamine were added and stirred for 1 h. After cooling to room temperature, the reaction solution was poured into ice water. The solid precipitate was filtered. Recrystallized from ethanol and dried to obtain compound Bibc-OH (69.8%). ^1^H NMR (600 MHz, DMSO) δ 13.47 (s, 1H), 8.55 (d, J = 2.1 Hz, 1H), 8.04–7.93 (m, 4H), 7.86 (dd, J = 8.6, 2.0 Hz, 1H), 7.74 (dd, J = 5.6, 3.3 Hz, 2H), 7.36 (dd, J = 6.0, 3.1 Hz, 2H), 7.20 (d, J = 8.6 Hz, 1H). ^13^C NMR (151 MHz, DMSO) δ 158.98, 151.21, 144.12, 133.38, 129.62, 127.24, 125.78, 123.91, 118.48, 113.14, 109.91. HRMS (EI) *m*/*z* calculated for [C_20_H_13_N_3_O + H]+: 312.1137, Found: 312.1117 (Figures S14–S16).

### 3.3. Synthesis of Bibc-DNBS

Bibc-OH (31.1 mg, 0.1 mM), 2,4-dinitrobenzenesulfonyl chloride (40.0 mg, 0.15 mM), and 28.0 μL of triethylamine were dissolved in a 50 mL round-bottomed flask containing 10.0 mL of dichloromethane, which was stirred for 3 h at room temperature. The reaction solution was extracted with dichloromethane (3 × 40 mL), followed by washing with saturated physiological saline. Subsequently, the organic phase was dried using anhydrous sodium sulfate. The resultant mixed liquid was distilled under evaporation to remove the solvent. Ultimately, column chromatography purification was conducted using a dichloromethane-to-ethyl acetate solvent system (30:1), yielding Bibc-DNBS with a yield of 72.9%. ^1^H NMR (600 MHz, DMSO) δ 12.83 (s, 1H), 8.64 (d, J = 1.9 Hz, 1H), 8.27 (d, J = 1.9 Hz, 1H), 8.15 (dd, J = 8.6, 2.0 Hz, 1H), 8.05 (d, J = 8.3 Hz, 3H), 8.01 (d, J = 8.3 Hz, 2H), 7.97 (d, J = 8.7 Hz, 1H), 7.67 (d, J = 8.6 Hz, 1H), 7.41 (d, J = 8.6 Hz, 2H), 7.13 (s, 2H). ^13^C NMR (151 MHz, DMSO) δ 150.36, 147.50, 146.80, 142.88, 138.98, 133.52, 128.42, 125.64, 120.25, 119.17, 111.44. HRMS (EI) *m*/*z* calculated for [C_26_H_15_N_5_O_7_S − H]+: 540.0614, Found: 540.0648 (Figures S17–S19).

### 3.4. Spectral Studies

The stock solution was obtained by dissolving a certain amount of probe Bibc-DNBS (1.5 mM) in DMSO. Bibc-DNBS stock solution (10.0 μM) and other analytes (100.0 μM) including Cys, Hcy, GSH, Phe, Ala, Trp, His, Lys, Pro, Met, Arg, Asp, Glu, Thr, Val, Ile, Mn^2+^, Na^+^, Mg^2+^, AcO^−^, NO_2_^−^, SO_3_^2−^, PO_4_^3−^, glucose, and citric acid were mixed separately. The mixture was diluted with PBS buffer (containing 20% acetonitrile, pH 7.4) to a final volume of 3.0 mL. β-lactamase inhibitors clavulanic acid and tazobactam were dissolved in deionized water to prepare a stock solution at a concentration of 10.0 mM. Dilute β-lactamase to 10^7^ U/mL with PBS buffer and stand by. The mixture of ethanol and PBS (10.0 mM, pH = 7.4, *v*:*v* = 2:8) was used to fix the volume of β-lactamase.

### 3.5. Cells and Zebrafish Imaging

HepG2 cells were cultured in DMEM medium supplemented with 10% fetal bovine serum and 100.0 U/mL penicillin–streptomycin solution in a humidified incubator with 5% CO_2_ at 37 °C. Prior to imaging, the cells were seeded onto a laser confocal culture dish at a density of 2 × 10^5^ per well. Following adhesion, the cells were washed with PBS three times. For the control group, HepG2 cells were co-cultured with Bibc-DNBS for 30 min at 37 °C. In the experimental group, HepG2 cells were pretreated with N-ethylmaleimide (NEM) for 30 min, then incubated with Cys, Hcy, and GSH (100.0 μM) for 30 min, respectively, and finally treated with Bibc-DNBS for 30 min. For the oxidative stress group, HepG2 cells were pretreated with LPS (100.0 μg/mL) for 0, 20, 40, and 60 min before being treated with probe Bibc-DNBS for 30 min, respectively. All treatment groups were subjected to confocal fluorescence imaging.

To image zebrafish, 4-day-old zebrafish were first cultured in E2 medium during a 12 h light/dark cycle. Zebrafish were merely treated with Bibc-DNBS (10.0 μM) for 30 min as control. The other zebrafish was pretreated by NEM (1.0 mM) for 30 min and then incubated with Bibc-DNBS (10.0 μM) for another 30 min. After removing the residue with PBS, confocal fluorescence imaging was performed. All animal-related experimental manipulations strictly followed the rigorous review and formal approval by the Animal Ethics Committee of Qiqihar Medical University (approval number QMU-AECC-2024-111).

### 3.6. Drug Resistance Experiment

Beyond cefazolin sodium as a substrate, our research now includes four additional antibiotics that exhibit diverse structural features: cefotiam, cefonicid, cefamandole, and ceftriaxone were selected to evaluate the hydrolysis rate of antibiotics by β-lactamases. Pipette 25.0 μL of the antibiotic solutions into 96-well plates, add 10.0 μL of β-lactamase (0.4 U/mL) and the appropriate amount of PBS buffer for incubation, and then add Bibc-DNBS to the volume of 200.0 μL/well. The test system was shaken and recorded with a multifunctional enzyme labeler (λ_ex_ = 310 nm, λ_em_ = 462 nm).

### 3.7. Bacteriostatic Experiment

*Enterobacter cloacae* ATCC 13047 and *Staphylococcus aureus* ATCC 29213 lyophilized powder were activated. After being cultured for 24 h in a full-temperature shaking incubator, the two strains were inoculated into fresh TSB medium, respectively. The diluted strain was spread evenly throughout the tryptic casein soybean peptone solid medium. Paper sheets loaded with drugs (1.0 μg for cefotiam, cefonicid, cefamandole, ceftriaxone, and cefazolin sodium) were placed on the solid medium and incubated at a constant temperature (15 h); photographs were taken and recorded.

## 4. Conclusions

In summary, we designed and synthesized a novel fluorescent probe, Bibc-DNBS, for the detection of biothiols and the indirect detection of β-lactamases. Bibc-DNBS displayed a large Stokes shift and a powerful fluorescence response and was successively applied to the detection of biothiols in HepG2 cells and zebrafish. It was confirmed by HRMS and HPLC that the 2,4-dinitrobenzenesulfonyl moiety of Bibc-DNBS was removed to produce the fluorophore Bibc-OH. Bibc-DNBS is easily synthesized and possesses high sensitivity, making it a promising candidate as a tool for screening β-lactamase inhibitors. It also demonstrates significant potential in the treatment of bacterial infections and resistance-related diseases.

## Data Availability

The data presented in this study are available in the Supplementary Materials.

## Supplementary Materials

The following supporting information can be downloaded at https://www.mdpi.com/article/10.3390/ijms26020525/s1.
