# Supplementary material for: Advanced Large-Stokes-Shift Fluorescent Probe for the Detection of Biothiols: Facilitating Accurate Indirect Measurement of β-Lactamases
Source: Int J Mol Sci. 2025 Jan 9;26(2):525. doi: 10.3390/ijms26020525 (PMC11764503; doi:10.3390/ijms26020525)
Supplement: Supplementary file 1 [file ijms-26-00525-s001.zip › ijms-3409526-supplementary.pdf]

Supporting information for

Advanced Large Stokes Shift Fluorescent Probe for the Detection of BioThiols: Facilitating Accurate Indirect Measurement of β-Lactamases

Likun Liu[a], Dongling Yan[b], Yukun Ma[a], Peng Hou[b], Pengfei Qi[b], Xue Zhang[b], Yitong Liu[b], Song Chen[b,*]

[a] Research Institute of Medicine & Pharmacy, Qiqihar Medical University, Qiqihar, 161006, PR China

[b] College of Pharmacy, Qiqihar Medical University, Qiqihar, 161006, PR China

[*]Corresponding author,

E-mail address: chensongchemistry@163.com

Table S1......S2

Figures S1-2...... S5

Figures S3-5...... S6

Figures S6-8...... S7

Figures S9-11...... S8

Figures S12-13...... S9

Figures S14-15...... S10

Figures S16-17...... S11

Figures S18-19...... S12

Table S2......S13

Table S1. Comparison of the proposed probe with other reported fluorescence probes for the detection of biothiols.

Probe	Stokes shift	Solvent (pH=7.4)	Detection limit	Response time	Applications	Literature
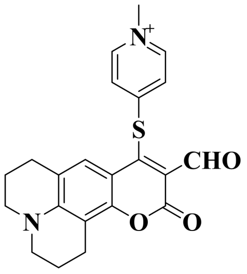	104nm/61nm	PBS	Cys(132nM)<br>Hcy(105nM)<br>GSH(62nM)	Cys (190s)<br>Hcy (155s)<br>GSH (80s)	HeLa cells, Zebrafish	Dyes Pigments 2022, 199, 110058
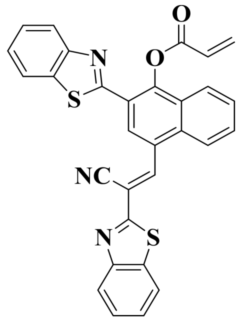	107nm	PBS/DMSO (3/7,v/v)	Cys(0.076μM)	Cys (15min)	PC3 cell, Mice	Journal of Photochemistry & Photobiology, A: Chemistry 2023, 436, 114383
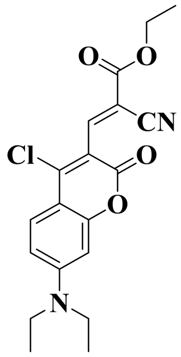	Hcy(78nm)<br>Cys(80nm)<br>GSH(81nm)	PBS/DMSO (7/3,v/v)	Hcy(3nM)<br>Cys(6nM)<br>GSH(200nM)	15min	A375 cells	Talanta 2020, 219, 121353
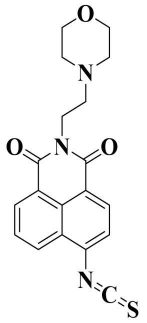	140nm	PBS/DMSO (8/2,v/v)	Cys(16.3nM)	50min	HepG2 cells, Zebrafish, Arabidopsis thaliana, Paper, Food, Water	Spectrochimica Acta Part A: Molecular and Biomolecular Spectroscopy 2024, 304, 123345

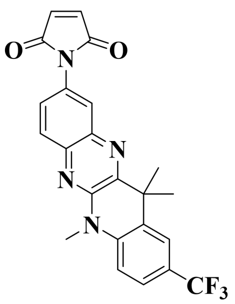	95nm	DMSO/PBS (1/9,v/v)	Cys(36.6nM) Hcy(116.5nM) GSH(57.1nM)	Cys (30min) Hcy (180min) GSH (180min)	HeLa cells, Zebrafish	Tetrahedron 2023,139, 133433
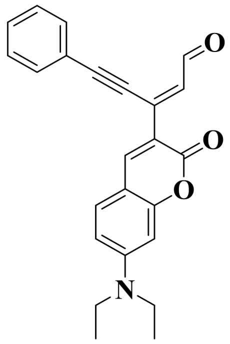	Cys/Hcy (66nm) GSH (81nm)	DMSO/PBS (1/1,v/v)	Cys(36.6nM) Hcy(86.4nM) GSH(174nM)	<1h	HepG2 cells	Spectrochimica Acta Part A: Molecular and Biomolecular Spectroscopy 2023, 292, 122410
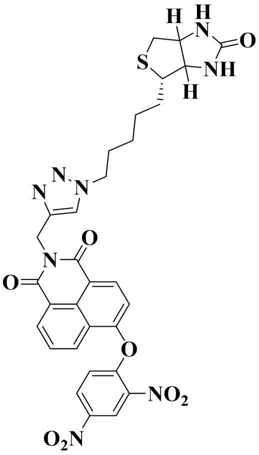	120nm	DMF/PBS (3/7,v/v)	2.7×10^{-8}M	------	HepG2 cells, Cancer cells, Zebrafish, Inflammation	Journal of Photochemistry & Photobiology, A: Chemistry 2023, 444, 114919
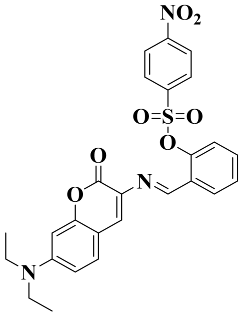	110nm	DMF/H_2O (1/9,v/v))	Cys(0.236μM) Hcy(0.365μM) GSH(0.223μM)	<3min	HeLa cell	Dyes Pigments 2022, 208, 110762

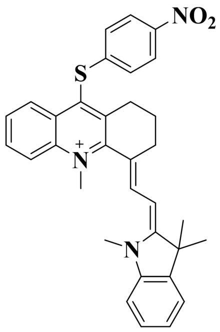	166nm	PBS/DMSO (7/3,v/v)	Cys(32nM) Hcy(345nM) GSH(205nM)	Cys (15min) Hcy/GSH (20min)	A549 cells, HepG2 cells	Sensors & Actuators: B. Chemical 2024, 414, 135994
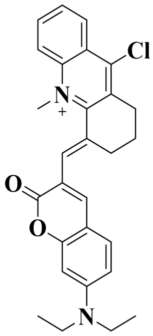	194nm	DMSO/PBS (1/1,v/v)	Cys(46nM) Hcy(83 nM)	Cys (3min) Hcy (15min)	A549 cell, Mice	Sensors and Actuators: B. Chemical 2023, 374, 132799
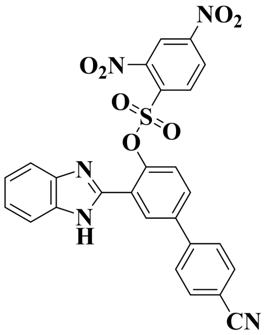	**172nm**	**PBS/CH$_3$N (8/2,v/v)**	**Cys(0.042μM) Hcy(0.092μM) GSH(0.121μM)**	**300s**	**HepG2 cell, Zebrafish, Indirect detection of β-Lactamase, β-lactamase inhibitor screening, Drug Resistance**	**This work**

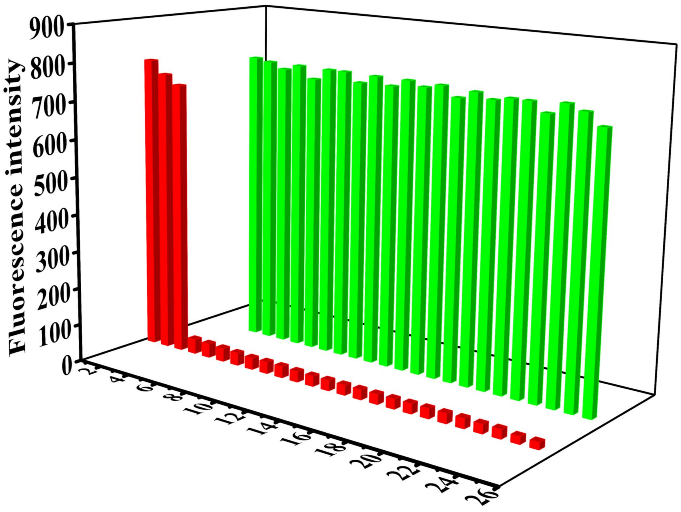

Figure S1 Changes in fluorescence intensity of the fluorescent probe Bibc-DNBS. (Red) Fluorescent probe Bibc-DNBS (10.0 μM) interacted with different analytes (100 μM)(1-25: Cys, Hcy, GSH, Asp, Ala, Trp, His, Ile, Arg, Met, Pro, Phe, Glu, Thr, Val, Na^+, Mn^{2+}, Lys, Mg^{2+}, AcO^-, NO_2^-, SO_3^{2-}, Glucose, Citric acid). (Green) In the presence of interferences (4-25: Asp, Ala, Trp, His, Ile, Arg, Met, Pro, Phe, Glu, Thr, Val, Na^+, Mn^{2+}, Lys, Mg^{2+}, AcO^-, NO_2^-, SO_3^{2-}, PO_4^{3-}, Glucose, Citric acid), Changes in fluorescence intensity of Bibc-DNBS (10.0 μM) react with Cys (100.0 μM).

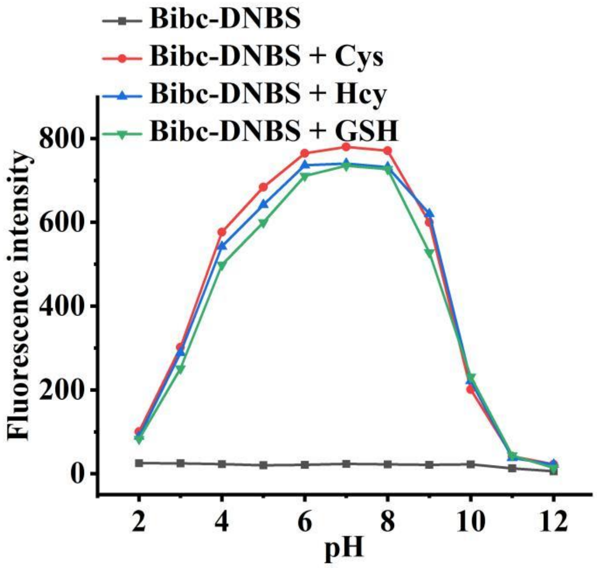

Figure S2 Effect of pH (2.0-11.0) on the fluorescence intensity of the **Bibc-DNBS** (10.0 μM) at 462 nm in the absence/presence of Cys/Hcy or GSH (100.0 μM).

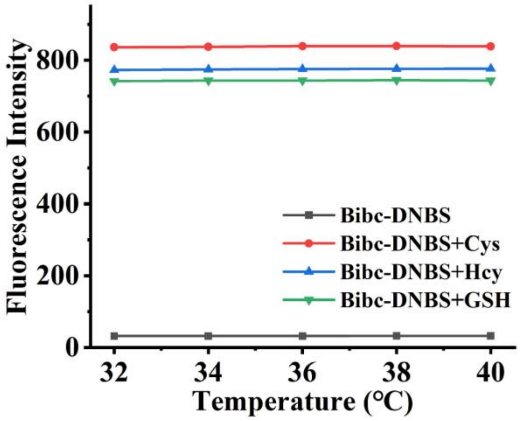

Figure S3 Effect of different temperatures on the detection of Cys/Hcy/GSH (100.0 μM) by the probe Bibc-DNBS (10.0 μM).

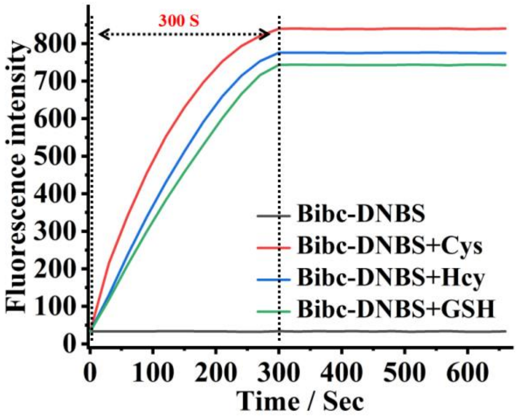

Figure S4 Time-dependent curves of probe **Bibc-DNBS** before and after reaction with Cys/Hcy/GSH (100.0 μM).

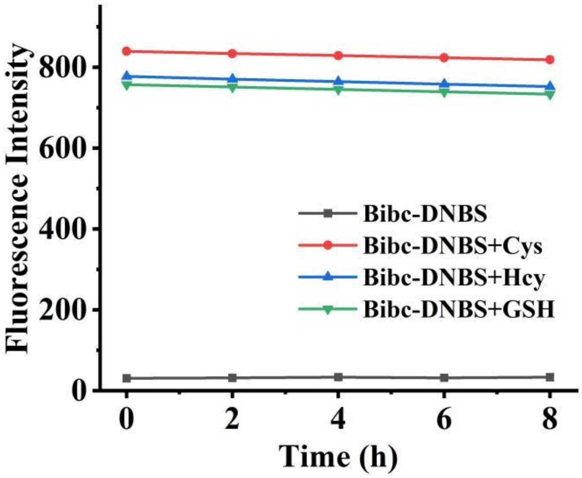

Figure S5 Stability assessment of the probe Bibc-DNBS (10.0 μM) for the detection of Cys/Hcy/GSH (100.0 μM) over 0-8 h.

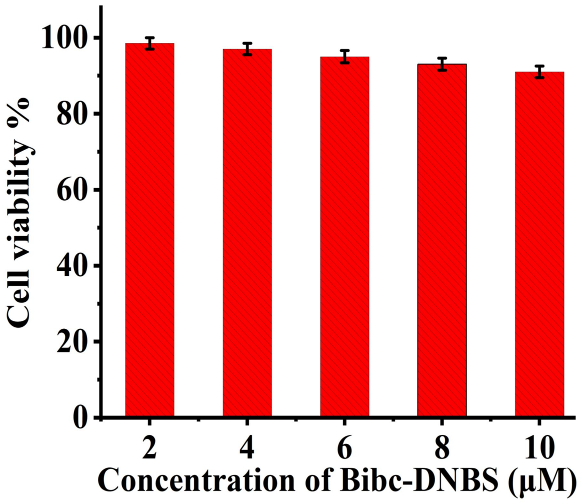

Figure S6 Survival rate of HepG2 cells treated with different concentrations of probe **Bibc-DNBS**.

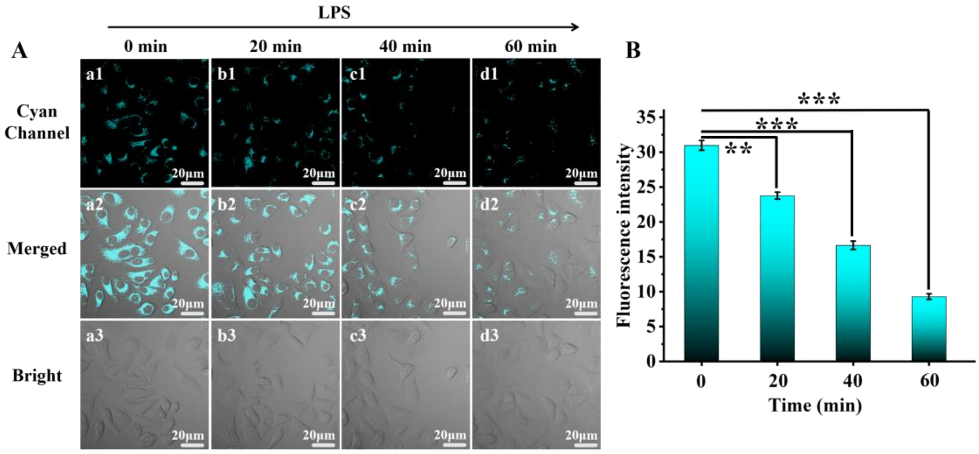

Figure S7. (A) Detection of thiol fluctuations during oxidative stress in HepG2 cells by probe Bibc-DNBS confocal imaging. (a-d) HepG2 cells treated with 100.0 μg/mL of LPS for 0-60 min, followed by treatment of probe Bibc-DNBS (10.0 μM) for another 30 min. (B) Fluorescence intensities in panel a-d. n = 3, error bars were ±SD. Statistical analysis was performed with a one-way ANOVA. Among them, **$p<0.01$, ***$p<0.001$. Scale bar 20 μm.

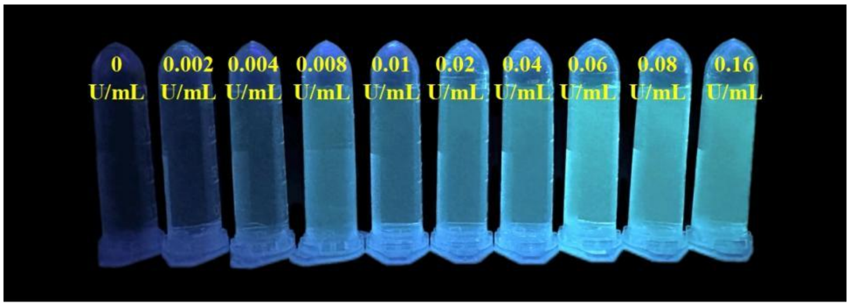

Figure S8 Various concentrations of β-lactamase (0.0-0.16 U/mL) were added and the corresponding **Bibc-DNBS** photographs were taken with a UV lamp at 365 nm.

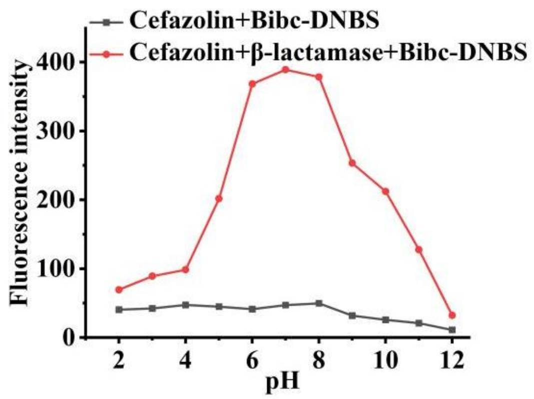

Figure S9 The fluorescence intensity of **Bibc-DNBS** at 462 nm at different pH values after incubation with cefazolin sodium (250.0 μM) and β-lactamase (0.02 U/mL) for 30 min.

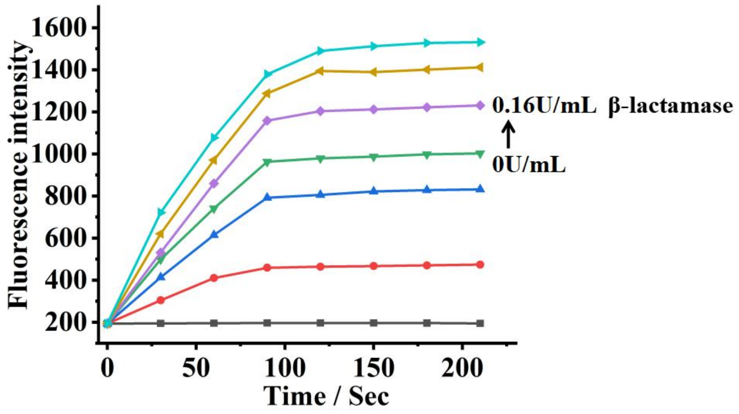

Figure S10 The fluorescence intensity of **Bibc-DNBS** (10.0 μM) was plotted against reaction time in the presence of different concentrations of β-lactamase (0, 0.005, 0.01, 0.02, 0.04, 0.08, 0.16 U/mL) using cefazolin sodium (250.0 μM) as substrate (using an enzyme-labeled instrument).

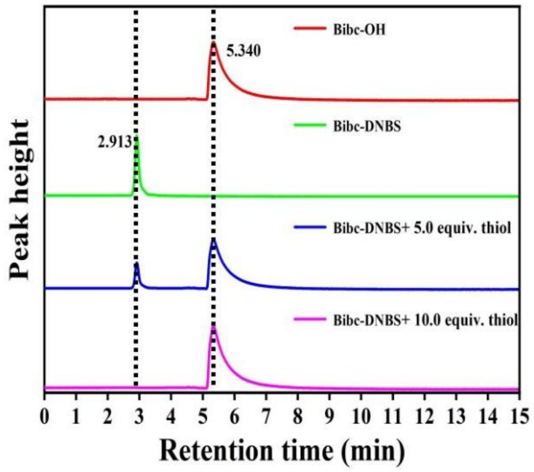

Figure S11 The HPLC chromatograms: (green) **Bibc-DNBS**; (blue/purple) **Bibc-DNBS** with gradually increasing amount (5.0, 10.0) equiv. of Cys incubated; (red) **BIbc-OH**. Condition: eluent, H_2O/CH_3CN (v/v, 9/1), flow rate, 1.0 ml/min; temperature, 34 °C; injection volume, 60.0 μL.

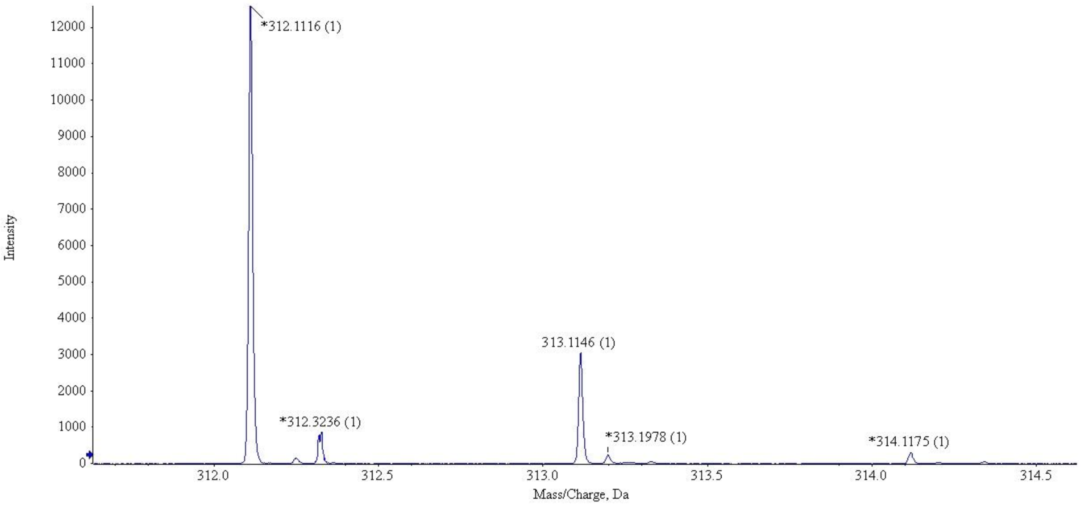

Figure S12 Mass spectrum of **Bibc-DNBS+Cys**

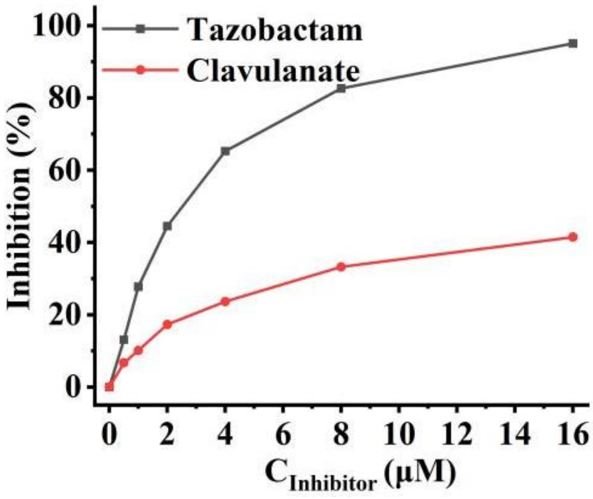

Figure S13 Determination of IC_{50} of *β*-lactamase by tazobactam and clavulanate.

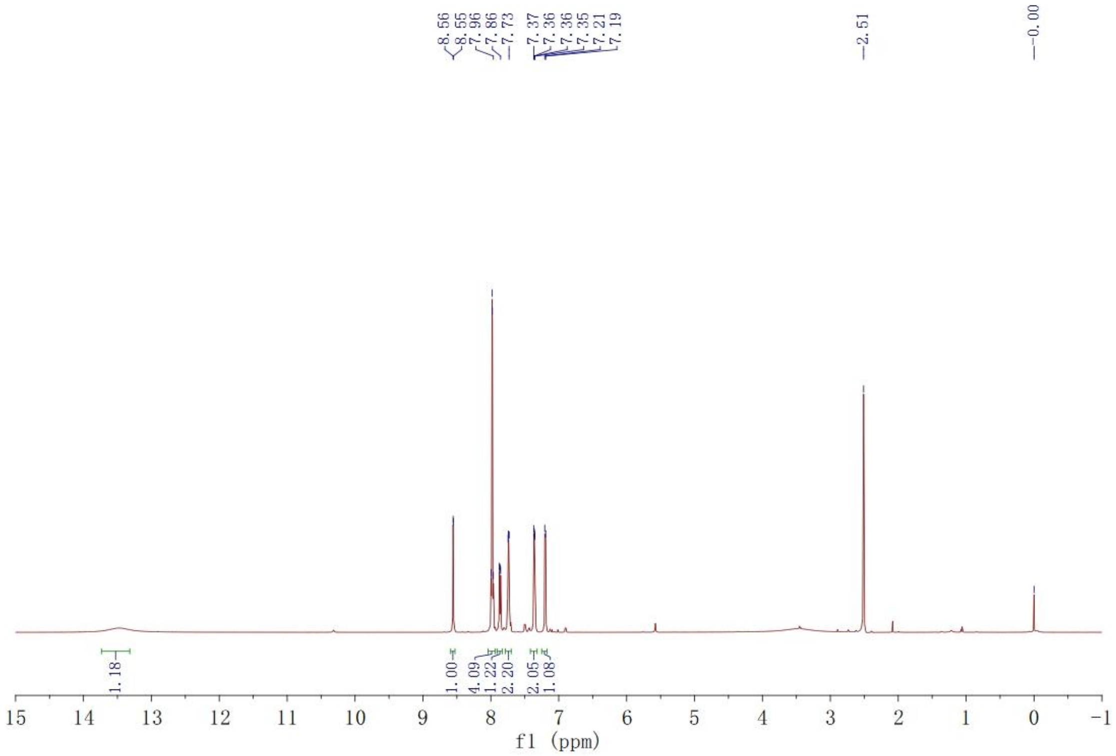

Figure S14 ^{1}H NMR spectrum of **BIbc-OH** in DMSO-d_6.

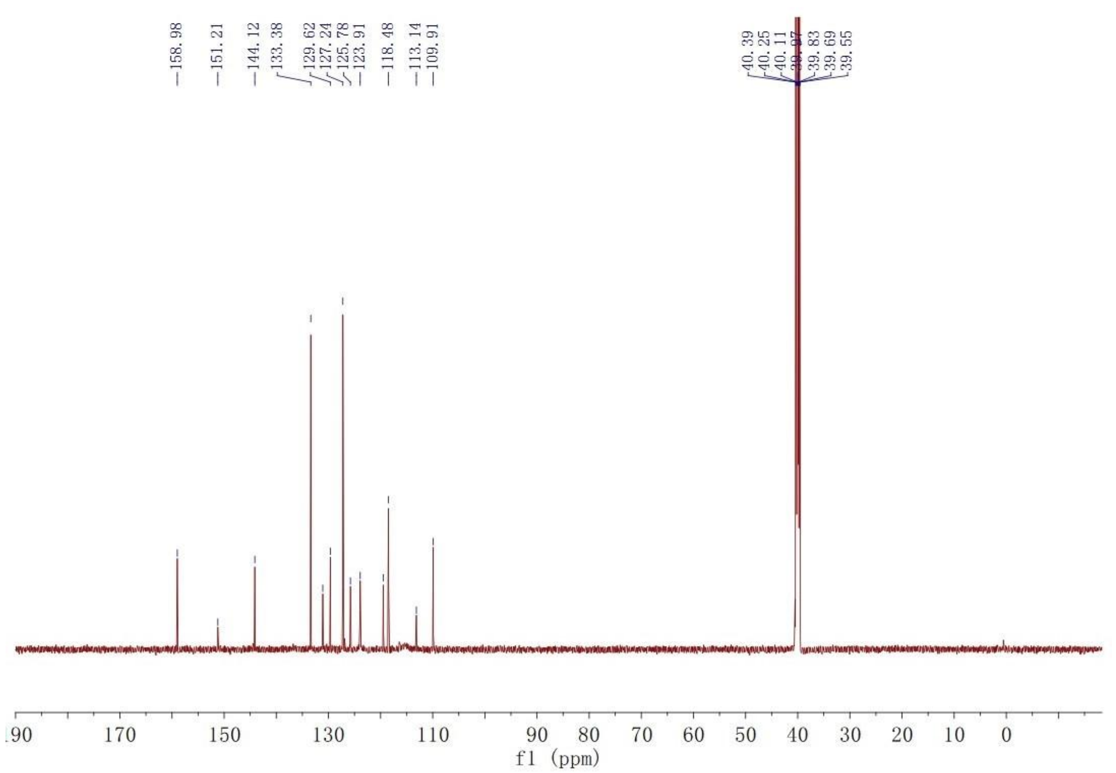

Figure S15 ^{13}C NMR spectrum of **BIbc-OH** in DMSO-d_6.

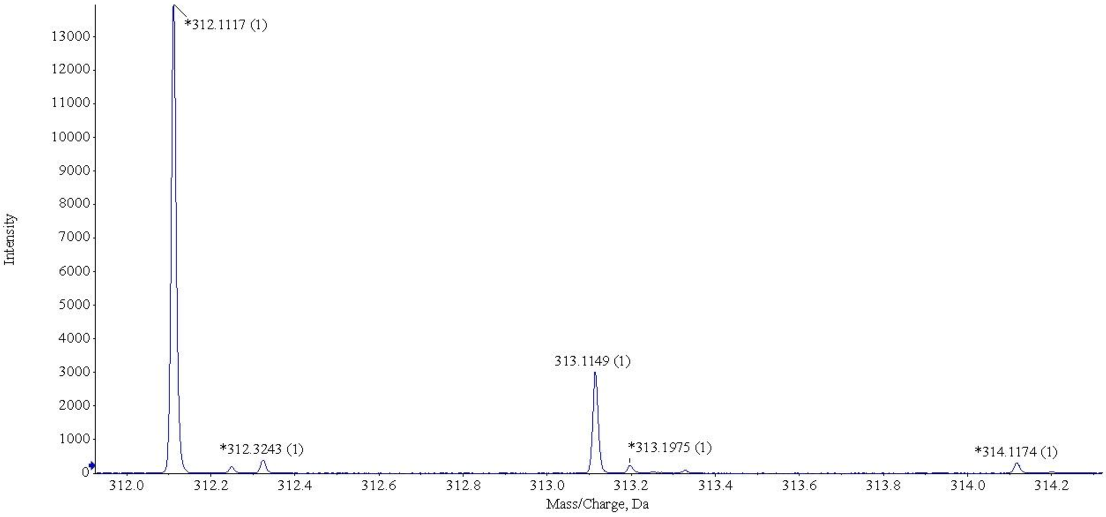

Figure S16 Mass spectrum of **BIbc-OH**.

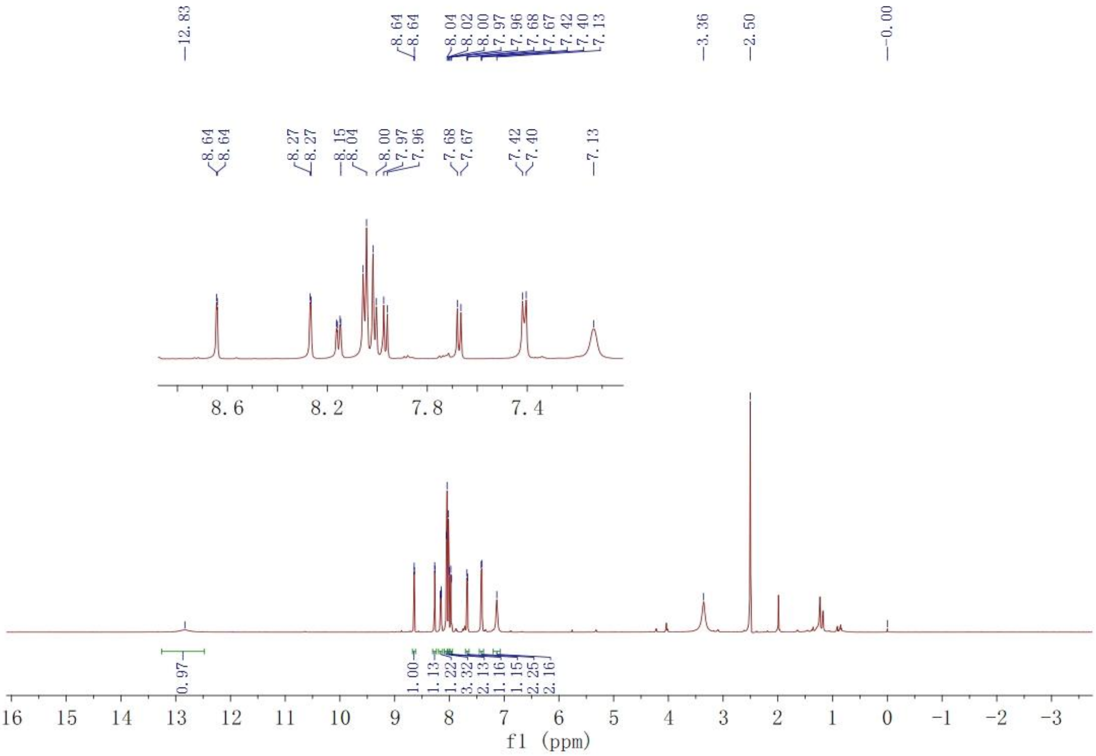

Figure S17 ¹H NMR spectrum of **Bibc-DNBS** in DMSO-d_6.

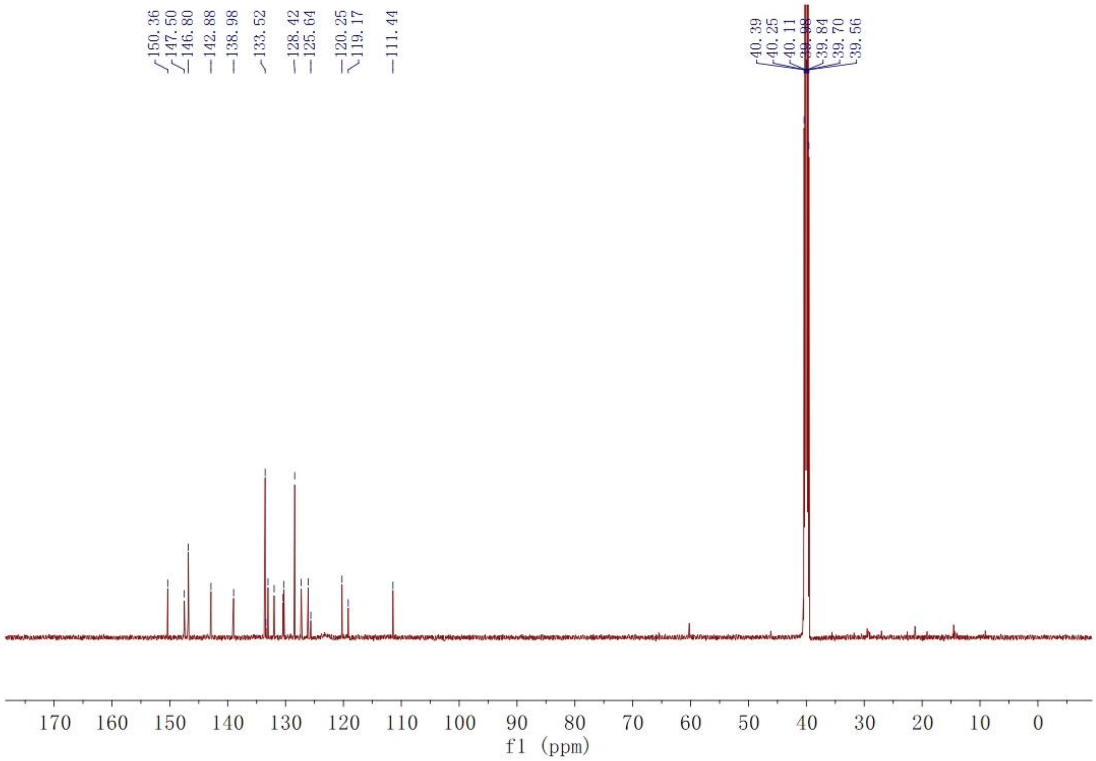

Figure S18 ^{13}C NMR spectrum of **Bibc-DNBS** in DMSO-d_6.

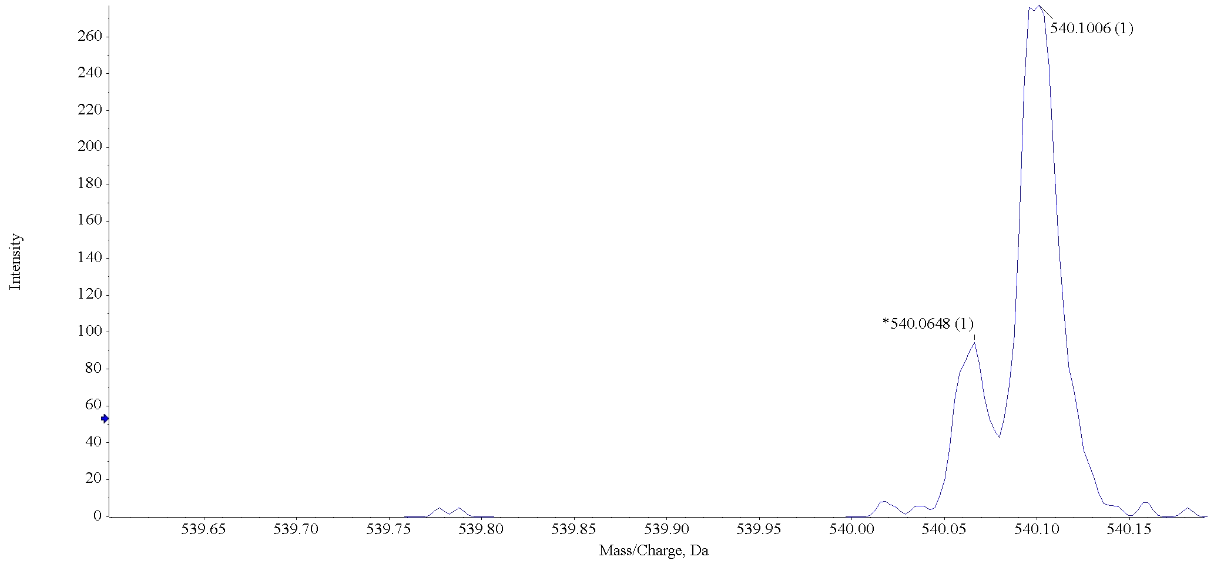

Figure S19 Mass spectrum of **Bibc-DNBS**.

Table S2. Comparison of key parameters of **Bibc-DNBS** with similar probes and different classes of probes

Probe Structure / Type	Detection item (Detection limit)	Stokes shift	Response time	Applications	Literature
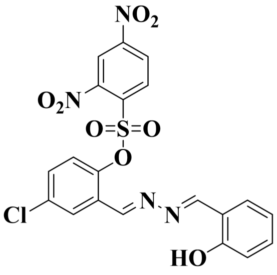	β-lactamase (0.5 mU/mL)	-	8 min	Test papers, Milk sample	Journal of Materials Chemistry B. 2018, 6, 3922
Cu(II) ions	Ampicillin	106 nm	60 min	-	Analytical Biochemistry 2005, 341, 113-121
Magnetic epitope-imprinted microsphere 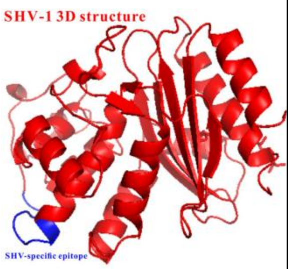	β-lactamases (2 μg/mL)	-	30 min	Bacteria	Journal of Nanobiotechnology 2024, 22, 678
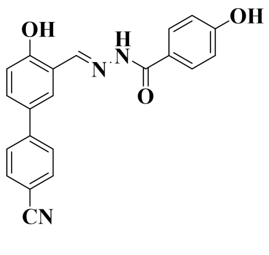	Al^{3+} (0.252 μM) Zn^{2+} (0.174 μM)	Al^{3+} (74 nm) Zn^{2+} (97 nm)	Al^{3+} (30min) Zn^{2+} (immediate)	Real water samples, Test papers, A549 cells	Spectrochimica Acta Part A: Molecular and Biomolecular Spectroscopy 2025, 329, 125538
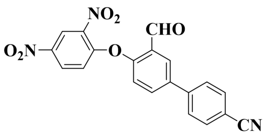	H_2S（0.18 μM）	183 nm	2 min	MDA-MB-231 cells	Spectrochimica Acta Part A: Molecular and Biomolecular Spectroscopy 2018, 203, 258-262

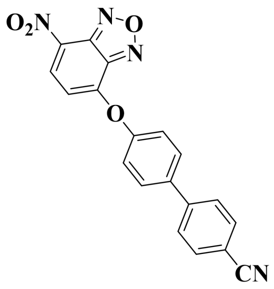	Cys(0.011 μM)<br>Hcy(0.015 μM)<br>GSH(0.003 μM)	110 nm	150 s	MCF-7 cells, tumor tissues, zebrafish	Frontiers in Chemistry 2022, 10, 856994
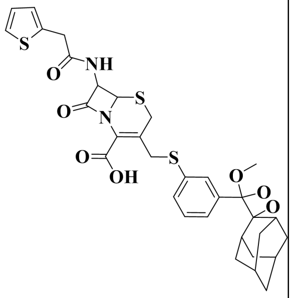	β-lactamase (5 nM)	-	20 min	E. coli	ChemComm 2020, 56, 3516
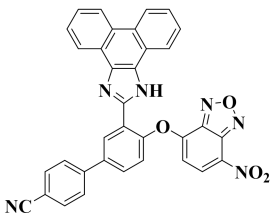	Cys(22.6 nM)<br>Hcy(31.2 μM)<br>GSH(17.7 μM)	105 nm	30 min	HeLa cells, Zebrafish	Bioorganic Chemistry 2019, 92, 103215
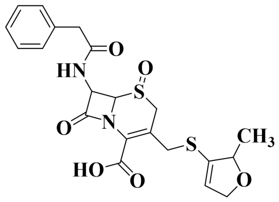	β-lactamase (0.2 nM)	-	30 min	Bacteria	Analytical Chemistry 2023, 95, 6098-6106
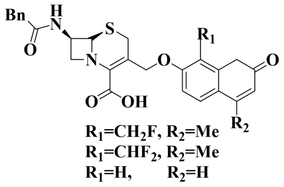	β-lactamase (0.5 nM)	95 nm	80 min	E. coli	COMMUNICATIO N 2016, 24, 3493-3497
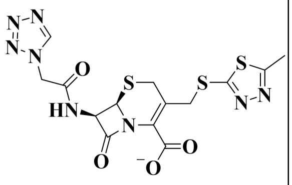	β-lactamase (0.5 mU mL^{-1})	193 nm	30 min	Test strip	Journal of Materials Chemistry B 2018, 6, 3922

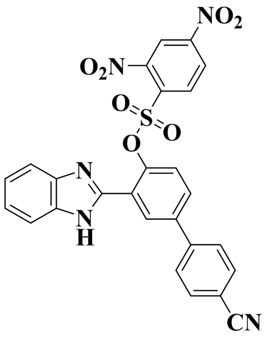	**Cys(0.042 μM) Hcy(0.092 μM) GSH(0.121 μM) β-lactamase (1.8×10^{-5} U/mL)**	**172 nm**	**300 s**	**HepG2 cell, Zebrafish, Indirect detection of β-Lactamase, β-lactamase inhibitor screening, Drug Resistance**	**This work**
